# Kras mutations increase telomerase activity and targeting telomerase is a promising therapeutic strategy for Kras-mutant NSCLC

**DOI:** 10.18632/oncotarget.10162

**Published:** 2016-06-18

**Authors:** Weiran Liu, Yuesong Yin, Jun Wang, Bowen Shi, Lianmin Zhang, Dong Qian, Chenguang Li, Hua Zhang, Shengguang Wang, Jinfang Zhu, Liuwei Gao, Qiang Zhang, Bin Jia, Ligang Hao, Changli Wang, Bin Zhang

**Affiliations:** ^1^ Department of Anesthesiology, Tianjin Medical University Cancer Institute and Hospital, National Clinical Research Center for Cancer, Key Laboratory of Cancer Prevention and Therapy, Tianjin, China; ^2^ Department of Lung Cancer, Tianjin Medical University Cancer Institute and Hospital, National Clinical Research Center for Cancer, Key Laboratory of Cancer Prevention and Therapy, Tianjin, China; ^3^ Department of radiotherapy, Tianjin Medical University Cancer Institute and Hospital, National Clinical Research Center for Cancer, Key Laboratory of Cancer Prevention and Therapy, Tianjin, China

**Keywords:** Kras mutations, lung cancer, telomerase, telomere, chemoresistance

## Abstract

As shortened telomeres inhibit tumor formation and prolong life span in a Kras^G12D^ mouse lung cancer model, we investigated the implications of telomerase in Kras-mutant NSCLC. We found that Kras mutations increased TERT (telomerase reverse transcriptase) mRNA expression and telomerase activity and telomere length in both immortalized bronchial epithelial cells (BEAS-2B) and lung adenocarcinoma cells (Calu-3). MEK inhibition led to reduced TERT expression and telomerase activity. Furthermore, telomerase inhibitor BIBR1532 shortened telomere length and inhibited mutant Kras-induced long-term proliferation, colony formation and migration capabilities of BEAS-2B and Calu-3 cells. Importantly, BIBR1532 sensitized oncogenic Kras expressing Calu-3 cells to chemotherapeutic agents. The Calu-3-Kras^G12D^ xenograft mouse model confirmed that BIBR1532 enhanced the antitumor efficacy of paclitaxel *in vivo*. In addition, higher TERT expression was seen in Kras-mutant NSCLC than that with wild-type Kras. Our data suggest that Kras mutations increase telomerase activity and telomere length by activating the RAS/MEK pathway, which contributes to an aggressive phenotype of NSCLC. Kras mutations-induced lung tumorigenesis and chemoresistance are attenuated by telomerase inhibition. Targeting telomerase/telomere may be a promising therapeutic strategy for patients with Kras-mutant NSCLC.

## INTRODUCTION

Lung cancer is a leading cause of cancer mortality worldwide [1]. Lung adenocarcinoma, the most common subtype of non-small cell lung cancers (NSCLC), always harbor a single mutated oncogene—the “driver” gene leading to cancer. Kras mutations are common driver mutations, and occur in approximately 20% of lung adenocarcinoma; point mutations in codons 12 or 13 are frequent [2–4]. Such point mutations result in constitutive activation of ras protein and impairment of GTPase activity, thereby activating the MAPK signaling pathway [5–7]. Many studies have shown that the Kras^G12D^ activating mutation induces abnormal cell proliferation and transformation, and initiates tumorigenesis in NSCLCs [8–11]. In mouse models of Kras^G12D^-driven NSCLCs, Kras^G12D^ expression promotes invasiveness and metastatic potential. It was reported that cancers with Kras mutations are insensitivity to anti-cancer drug treatments and patients with Kras-mutant NSCLC fail to benefit from chemotherapy [12–14]. Although Kras mutations were identified in NSCLC 20 years ago, effective therapies are still being pursued.

Telomeres are structures at the ends of linear chromosomes that protect chromosomes from degradation, irregular recombination and end-to-end fusions [15]. Telomeres shorten with each round of cell division, eventually resulting in cell crisis. A small subpopulation of cells bypass cell crisis and become immortal because of the reactivation of telomerase [16]. Telomerase, a reverse transcript enzyme, minimally composed of TERT and TR (telomerase RNA), synthesizes and maintains telomere [15]. Telomerase is silent in human normal somatic cells but is active in approximately 90% of cancers, including lung cancer, making it an attractive target for cancer therapy [17–19]. TERT transactivation is a key step for telomerase activity. Reportedly, activation of the Ras/MEK/ERK pathway upregulates telomerase by direct activating TERT transcription [20]. As Kras mutations result in continuous activation of the Ras/MEK/ERK pathway, Kras mutations may be involved in regulation of telomerase activity.

Telomerase activation and telomere attrition have been implicated in human lung tumorigenesis [21]. Telomere length shortens during the multistep human lung carcinogenesis progression until a stable short length is reached. In early neoplastic lesions, telomerase activity is low, which is not enough to counteract telomere shortening. In advanced stages of lung cancer, robust activation of telomerase can maintain telomere length stability, enabling the lung cancer to progress. The Kras^G12D^ lung cancer mice model with TERT deletion showed telomere dysfunction increased lung epithelial apoptosis, delayed tumor formation and increased life span [22]. These studies raise the possibility of targeting telomerase for Kras-mutant NSCLC therapeutics.

Here, we studied the functions of telomerase in Kras-mutant NSCLC. We found that Kras mutations transactivated TERT expression and increased telomerase activity and telomere length through activating the MEK/ERK pathway. Telomerase inhibition led to reduction of Kras mutations-induced cell long-term proliferation, cell colony formation and migration capabilities. In addition, telomerase inhibitor enhanced efficacy of paclitaxel. This study suggests potential use for telomerase inhibitors in Kras-mutant NSCLC therapeutics.

## RESULTS

### Kras mutations transactivate TERT expression and increase telomerase activity by activating the RAS-MEK pathway in both immortalized bronchial epithelial cells and lung adenocarcinoma cells

**Figure 1:**
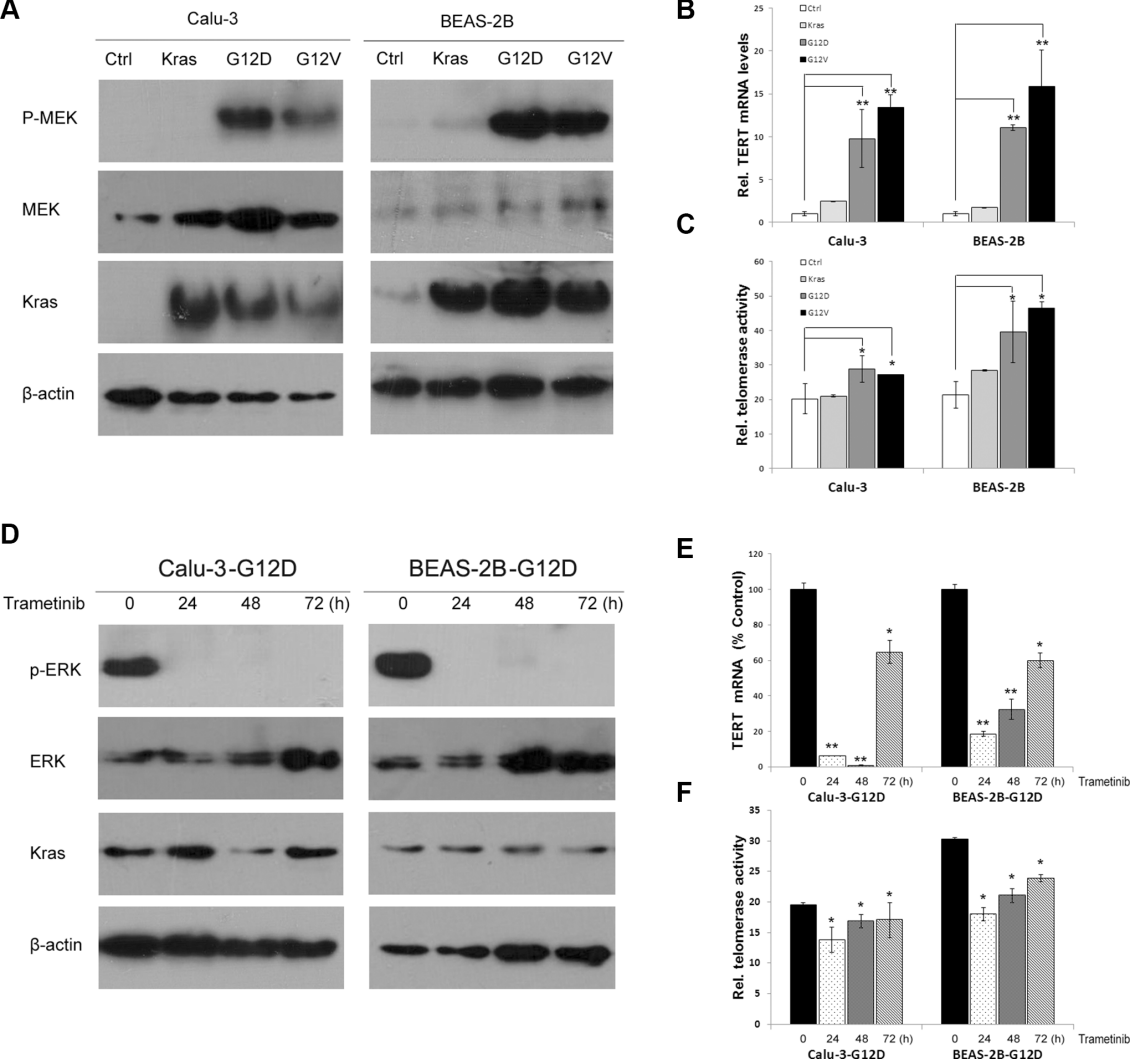
Kras mutations upregulate TERT expression and telomerase activities by the RAS-MEK pathway (**A**) Kras, Kras^G12D^, Kras^G12V^ and vector control were lentivirally transduced into BEAS-2B and Calu-3 cells for stable expression. Overexpression of Kras was confirmed by western blotting using Kras antibody. Kras^G12D^ and Kras^G12V^ activated Phospho–MEK. (**B**) Kras^G12D^ and Kras^G12V^ transactivated TERT mRNA expression by RT-qPCR analysis. (**C**) Kras^G12D^ and Kras^G12V^ elevated telomerase activity by a real-time quantitative TRAP assay. (**D**) MEK inhibitor trametinib inhibited phosphoralation of ERK in Kras^G12D^-overexpressing cells. Trametinib decreased TERT expression (**E**) and telomerase activity (**F**). Data were expressed as mean ± SEM (one-way ANOVA, *P < 0.05, **P < 0.01).

Previous studies have suggested that TERT is transactivated by the Ras/MEK/ERK pathway [20]. Because the Ras/MEK/ERK pathway is stimulated by Kras mutations, we first examined the effect of Kras mutations on TERT expression in cell lines. We transduced Kras, Kras^G12D^ and Kras^G12V^ with lentiviral expression vectors into immortalized human bronchial epithelial cells (BEAS-2B) and lung adenocarcinoma cells (Calu-3) for stable expression (Figure 1A). Kras^G12D^ and Kras^G12V^ overexpression significantly increased TERT mRNA expression in both cells compared with wild-type Kras-overexpressing cells and control cells (Figure 1B). Telomerase quantitative PCR analysis showed telomerase activities were also increased in Kras^G12D^ and Kras^G12V^-overexpressing cells (Figure 1C). Consistent with other reports, Kras mutations resulted in the activation of RAS-MEK pathway (Figure 1A). To investigate whether Kras mutations -induced TERT upregulation depended on the pathway, Kras^G12D^-overexpressing cells were treated with MEK inhibitor trametinib (Figure 1D). TERT expression (Figure 1E) and telomerase activities (Figure 1F) were significantly decreased after trametinib treatment. MEK siRNA-mediated MEK knockdown also reduced TERT mRNA levels in Kras^G12D^ -overexpressing cells (Supplementary Figure S1A and S1B). These findings strongly indicate that mutant Kras increased TERT expression and telomerase activities through Kras mutations-induced activation of the RAS–MEK pathway.

### Telomerase inhibitor BIBR1532 suppresses mutant Kras-induced proliferation of BEAS-2B and Calu-3 cells

**Figure 2:**
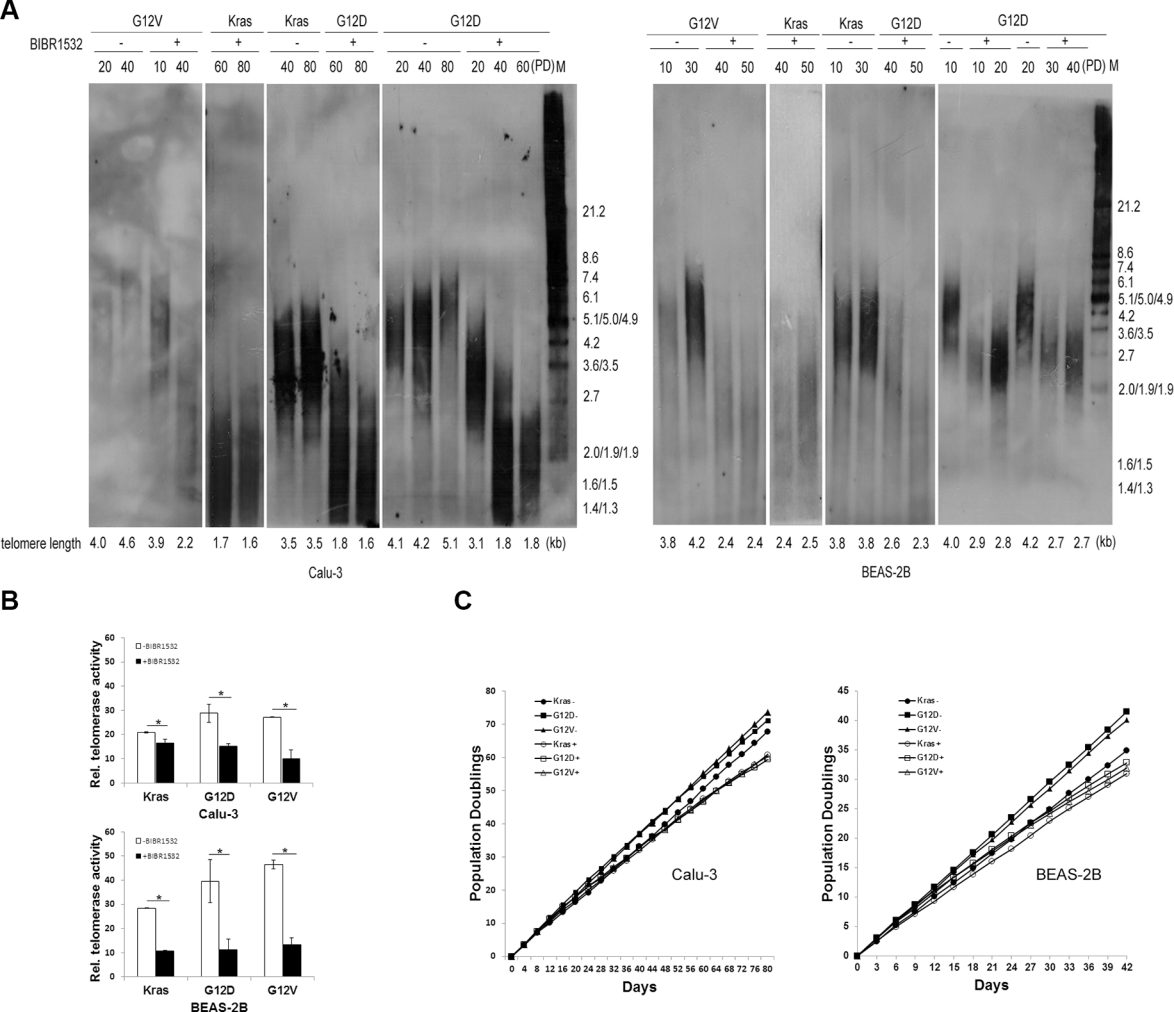
Telomerase inhibitor BIBR1532 shortens telomere length and suppresses mutant Kras-induced cell long-term proliferation in both BEAS-2B and Calu-3 cells (**A**) Cells were collected at the indicated population doublings and measured by TRF Southern blot analysis. Telomere length maintained stable in wild-type Kras expressing cells, but became long in the oncogenic Kras expressing cells with cell division. Continuous telomerase inhibitor BIBR1532 treatment resulted in telomere loss in both wild-type Kras and the oncogenic Kras expressing cells. (**B**) Real-time quantitative TRAP assay showed that telomerase inhibitor BIBR1532 inhibited telomerase activity of all cells. (**C**) Growth curve analysis of cells without (−) or with (+) 20 uM BIBR1532 domenstrated that BIBR1532 suppressed the oncogenic Kras-induced cell proliferation. PD: population doubling. one-way ANOVA, *p < 0.05.

Next, we detected whether Kras mutations lengthened telomeres. The terminal restriction fragment (TRF) length assay showed that telomere length was stable in wild-type Kras-overexpressing cells, but telomere length was gradually lengthened in Kras^G12D^ and Kras^G12V^-overexpressing cells with cell division (Figure 2A). When all the cells were treated with telomerase inhibitor BIBR1532, telomerase activities were decreased (Figure 2B). As expected, telomere length progressively shortened in both the oncogenic Kras expressing cells and wild-type Kras expressing cells after continuous BIBR1532 treatment (Figure 2A). And BIBR1532 led to telomere shortening in a dose dependent manner (Supplementary Figure S1C). However, BIBR1532 significantly decreased the oncogenic Kras-induced long-term cell proliferation in both BEAS-2B and Calu-3 cells (Figure 2C). These results indicate that continuous telomerase inhibition shortens telomeres length and suppresses mutant Kras-induced long-term cell proliferation.

### Telomerase inhibitor BIBR1532 suppresses mutant Kras-induced colony formation and migration of BEAS-2B and Calu-3 cells

**Figure 3:**
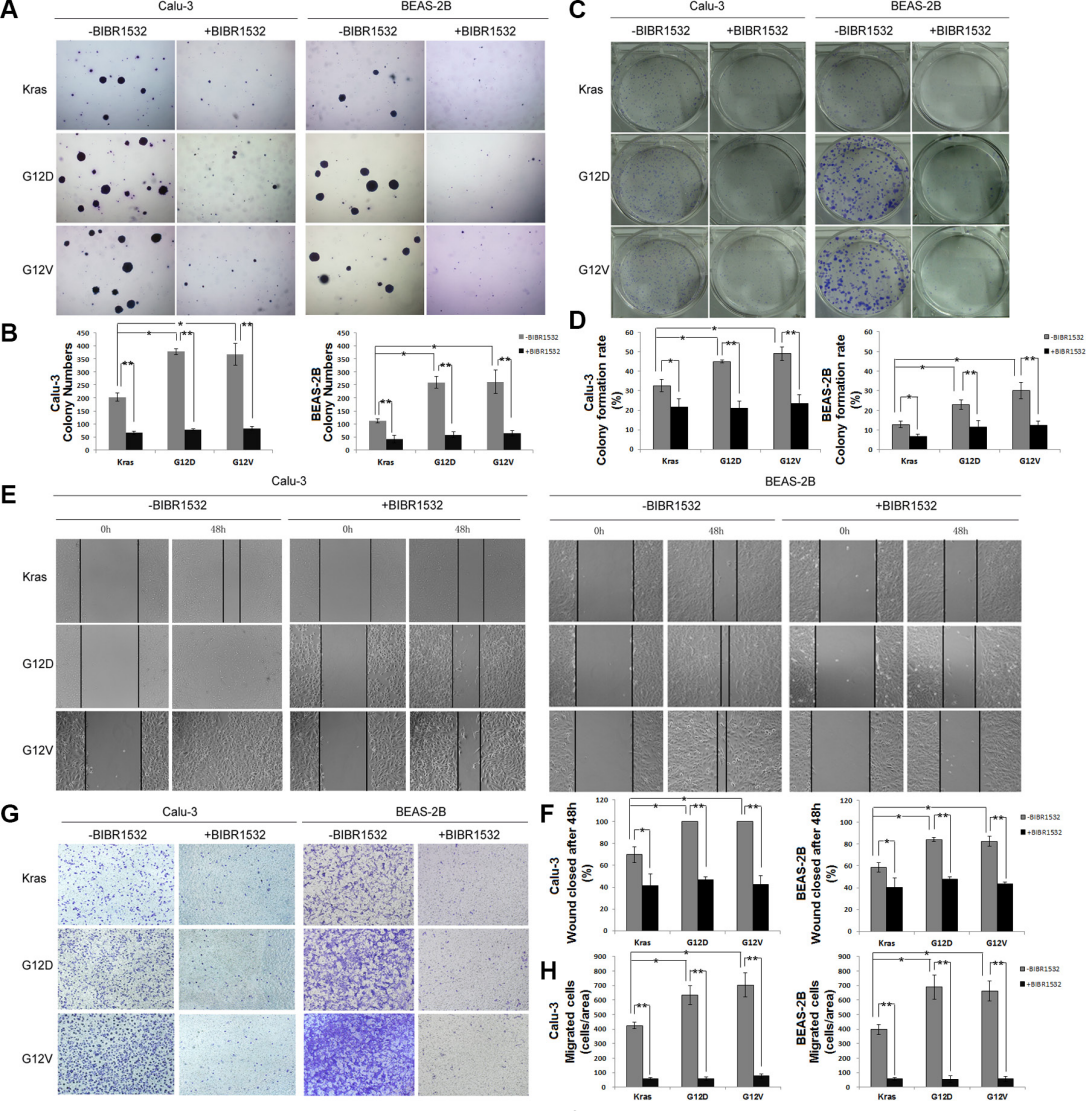
Telomerase inhibitor BIBR1532 inhibits Kras mutations-induced cell transformation and migration capability (**A**, **B**) Kras, Kras^G12D^ and Kras^G12V^-Calu-3 and -BEAS-2B cells were treated with or without BIBR1532 (20 uM) for 30 days before being replated in low melting point agarose. Colonies were allowed to grow for 20 days before being stained with crystal violet and counted. Photographs of crystal violet-stained colonies and colony numbers were shown. (**C**, **D**) The cells were plated in 6-well plates, treated with or without BIBR1532 (20 uM), and allowed to grow for 14 days to form clones. Clones with more than 50 cells were counted. (**E**, **F**) The cells were not treated or treated with BIBR1532 before wounds were made. Relative ratio of wound closure per field was shown. (**G**, **H**) Kras^G12D^ and Kras^G12V^-induced migration ability of lung cancer cells was inhibited by BIBR1532 in Transwell invasion assay. Numbers of invasive cells in 10 fields were counted. Magnification: ×200. Representative pictures were shown. Values were the mean of 3 determinations ± SEM (one-way ANOVA, *p < 0.05, **p < 0.01).

To examine the role of BIBR1532 on Kras mutations-induced anchorage-independent growth, Kras^G12D^ and Kras^G12V^-overexpressing BEAS-2B and Calu-3 cells were previously treated by BIBR1532 before being plated in low melting point agarose. BIBR1532 obviously inhibited the anchorage-independent proliferation and survival induced by activating Kras mutations in both BEAS-2B and Calu-3 cells (Figure 3A and 3B). Furthermore, oncogenic Kras-induced cell focus formation was dramatically reduced in both cell types after BIBR1532 treatments (Figure 3C and 3D). As BIBR1532 has been reported to have off-target effects in telomerase-negative cells, we confirmed the results with TERT shRNA. TERT shRNA was delivered into Kras^G12D^-overexpressing BEAS-2B and Calu-3 cells by lentivirus infection. We found TERT shRNA-mediated TERT knockdown also inhibited mutant Kras-induced anchorage-independent growth in soft agar and cell focus formation (Supplementary Figure S2).

To assess whether BIBR1532 suppressed oncogenic Kras-induced cell motility, we performed wound healing and Transwell migration assays in Kras^G12D^ and Kras^G12V^ -ovexpressing BEAS-2B and Calu-3 cells. We found closure of the wound was complete in Kras^G12D^ and Kras^G12V^-ovexpressing BEAS-2B and Calu-3 cells within 48h, but not in the same cells treated by BIBR1532 (Figure 3E and 3F). Transwell migration assay showed BIBR1532 profoundly inhibited oncogenic Kras-induced cell migration by both cell types (Figure 3G and 3H). Thus, we believe that telomerase inhibitor BIBR1532 suppresses Kras mutations-induced cell transformation and migration in NSCLC.

### BIBR1532 increased chemosensitivity of Kras^G12D^ and Kras^G12V^-overexpressing lung cancer cells

**Figure 4:**
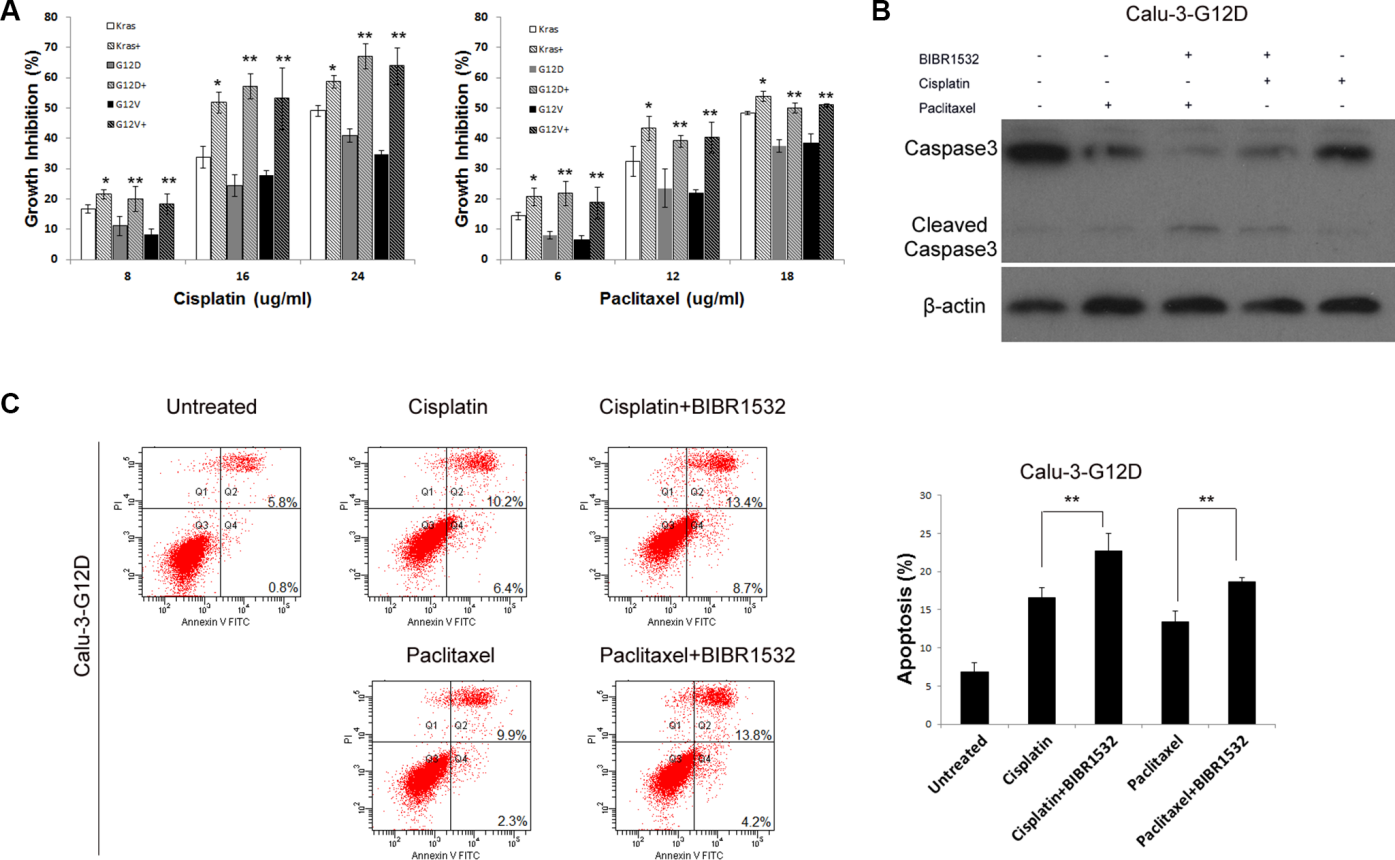
Telomerase inhibitor BIBR1532 increases sensitivities of Kras^G12D^ and Kras^G12V^-Calu-3 cells to cisplatin and paclitaxel (**A**) Cells were treated with or without BIBR1532 (20 uM) for 30 days before cisplatin and paclitaxel treatments. MTS assay showed that BIBR1532 significantly increased the sensitivity of Kras^G12D^ and Kras^G12V^-Calu-3 cells to cisplatin and paclitaxel. BIBR1532 enhanced cisplatin and paclitaxel cytotoxicity, as indicated by (**B**) increased caspase3 degradation and (**C**) AnexinV-PI apoptosis analysis. Data were expressed as mean ± SEM from three independent experiments (one-way ANOVA, *P < 0.05, **P < 0.01).

Cancers with Kras mutations often resist to anti-cancer drugs, so we tested whether telomerase inhibitor could overcome chemoresistance of Kras mutant lung cancer cells. As expected, overexpression of Kras^G12D^ and Kras^G12V^ indeed enhanced cell viability in response to cisplatin or paclitaxel treatment, compared with wild-type Kras-Calu-3 cells (Figure 4A). However, MTS assay showed that BIBR1532 increased the chemosensitivity of wild-type Kras-Calu-3 cells (Figure 4A). Furthermore, the effect of Kras^G12D^ and Kras^G12V^ in cisplatin or paclitaxel resistance was diminished in BIBR1532-treated Calu-3 cells (Figure 4A). Combined paclitaxel and BIBR1532 or cisplatin and BIBR1532 treatments also increased caspase3 degradation in Kras^G12D^-Calu-3 cells compared with paclitaxel- or cisplatin-only treatments (Figure 4B). AnnexinV-PI apoptosis analysis also confirmed that BIBR1532 enhanced paclitaxel or cisplatin-induced apoptosis (Figure 4C). These results show that BIBR1532 sensitized not only wild-type Kras-overexpressing but also oncogenic Kras-overexpressing lung cancer cells to chemotherapeutic agents.

### BIBR1532 enhanced paclitaxel's effect on Kras^G12D^-Calu-3 xenograft tumors in mice

**Figure 5:**
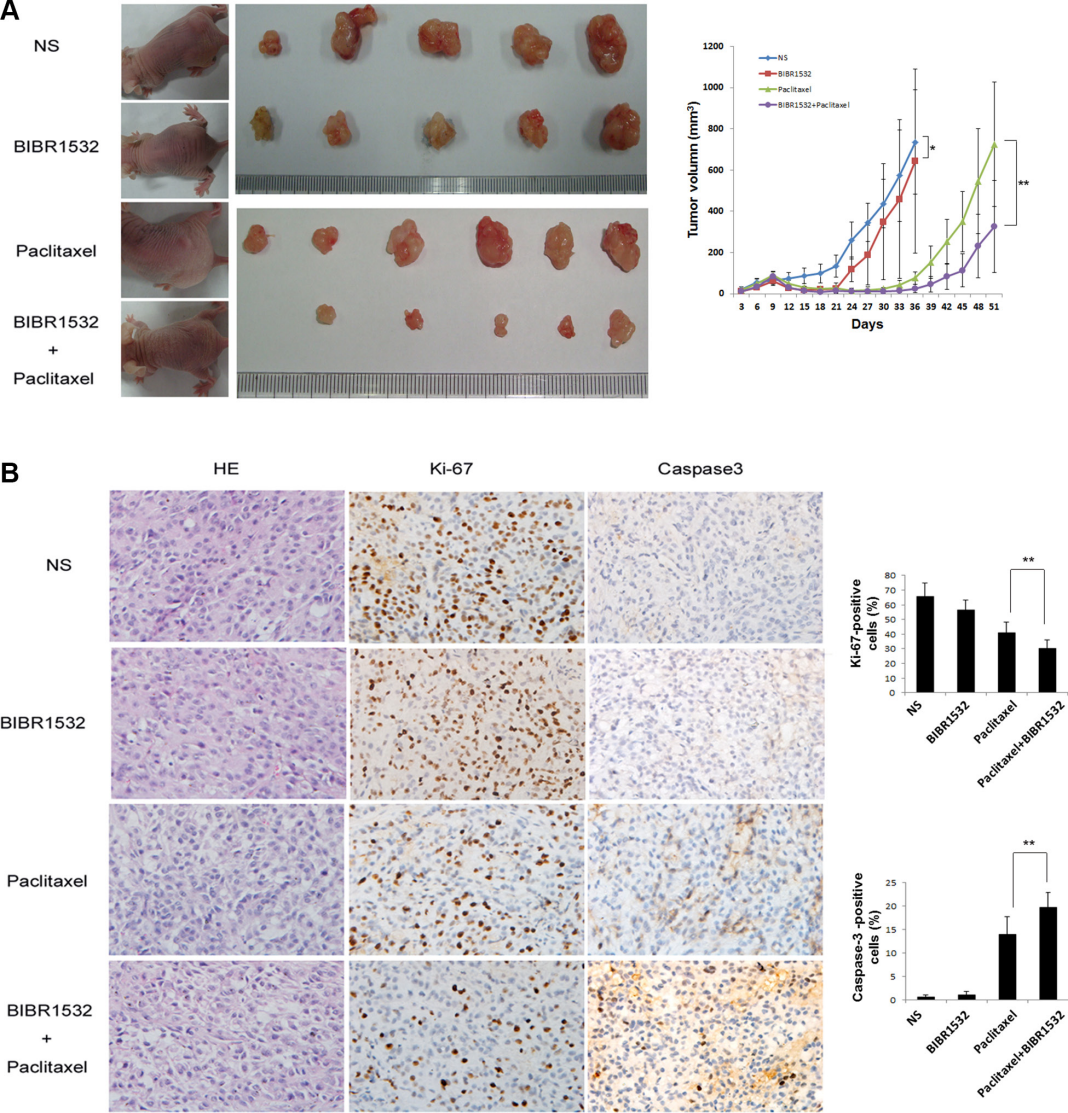
Telomerase inhibitor BIBR1532 prevents Kras^G12D^-Calu-3 xenograft tumor growth and increases the paclitaxel killing effect (**A**) Kras^G12D^-Calu-3 cells were pretreated with BIBR1532 and then injected subcutaneously into the flank of immunodeficient mice. When tumors grew to about 100 mm^3^, Kras^G12D^-Calu-3 xenografts mice were treated with normal saline, BIBR1532, paclitaxel, or BIBR1532 plus paclitaxel. Tumor grew more slowly in BIBR1532-treated group (*n* = 5) than NS-treated control group (*n* = 5), *p* < 0.05. BIBR1532 plus paclitaxel (*n* = 7) significantly decreased tumor growth compared with paclitaxel only (*n* = 7), *p* < 0.01. (**B**) HE stains showed tumor formation. Expression of Ki-67 and cleaved caspase-3 were measured by immunohistochemistry to evaluate proliferative and apoptotic ability of tumor cells. Representative images from each group were shown. The columns represented the mean ± SEM, one-way ANOVA, **p < 0.01.

Kras^G12D^-Calu-3 cells were injected into the flanks of immunodeficient mice. Treatment was initiated after appearance of palpable tumors. Mice were randomly divided into 4 groups; Group 1: controls treated with saline; Group 2: BIBR1532, every two days for 2 weeks; Group 3: paclitaxel, once a week for 2 weeks; and Group 4: combined BIBR1532 and paclitaxel. Compared with the untreated Group 1, tumors grew more slowly in the BIBR1532-treated Group 2 (Figure 5A). The paclitaxel + BIBR1532 Group 4 had better inhibition of tumor growth than did the paclitaxel only Group 3 (Figure 5A). Combined BIBR1532 and paclitaxel treatment also delayed tumor relapse. Tumor sections from these mice showed that paclitaxel moderately inhibited proliferation (Ki67^+^ cells) and enhanced apoptosis (cleaved caspase3^+^ cells) (Figure 5B), but the combined BIBR1532 and paclitaxel treatment significantly reduced the number of proliferative cells and increased the number of apoptotic cells (Figure 5B). The data indicate that BIBR1532 enhances the killing effect of paclitaxel. A combination of BIBR1532 and paclitaxel significantly blocked tumor growth in mice with Kras^G12D^-mutant lung cancer xenografts.

### TERT expression is higher in Kras^mut^ lung adenocarcinomas than in Kras^wt^ lung adenocarcinomas

**Figure 6:**
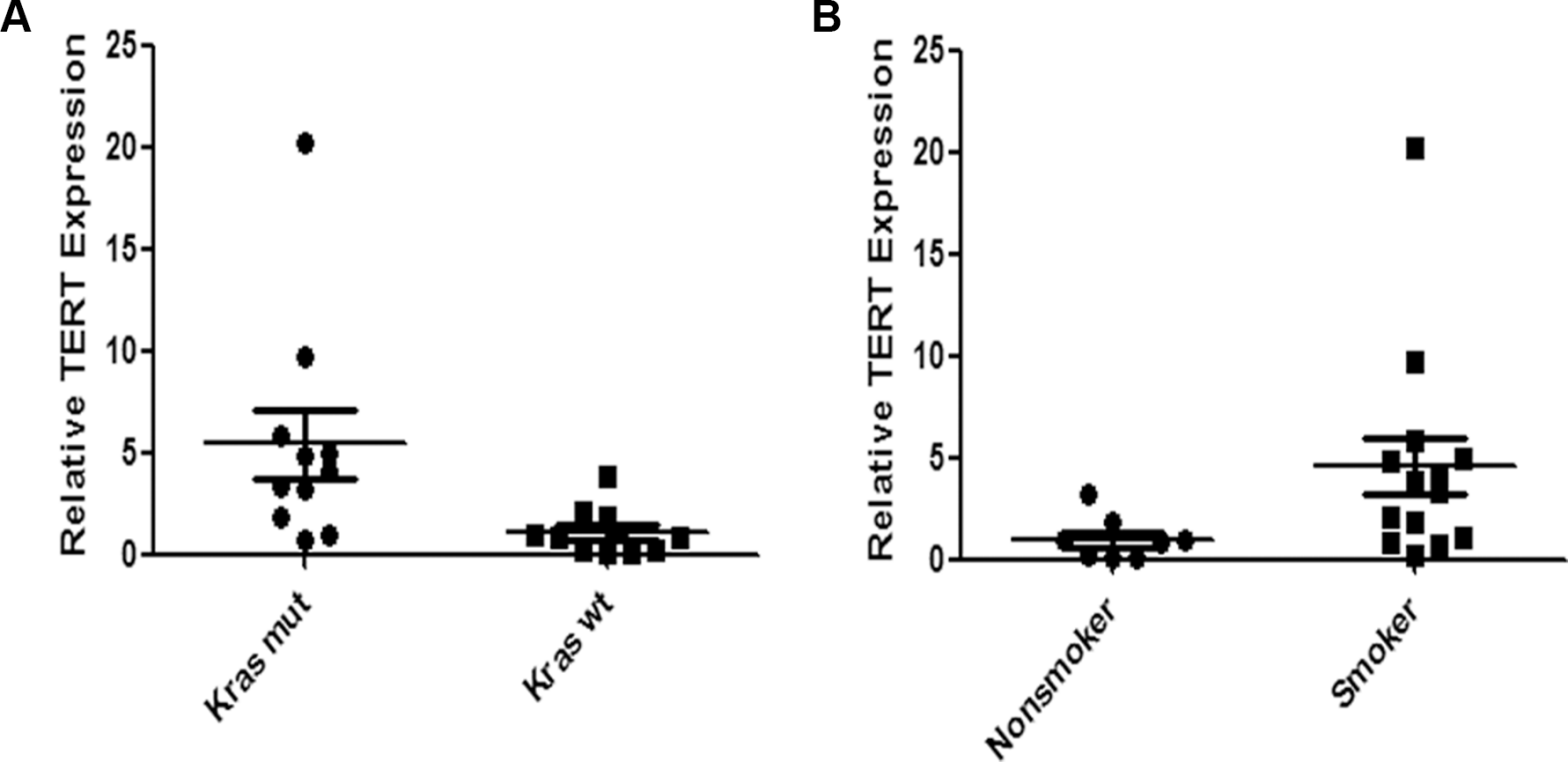
Expression of TERT mRNA is higher in Kras^mut^ lung adenocarcinoma than in Kras^wt^ lung adenocarcinoma We compared TERT mRNA expression between (**A**) lung cancer with Kras^mut^ and with Kras^wt^ (*P* = 0.0031) and (**B**) between smoker and non-smoker (*P* = 0.0127) by RT-qPCR. Differences were statistically analyzed using Mann Whitney test.

We examined TERT mRNA expression in surgical specimens from 22 lung adenocarcinoma patients according to Kras status using quantitative RT-PCR assay. There were 11 Kras^mut^ cases and 11 Kras^wt^ cases in the specimens. Patients' clinicopathological characteristics are summarized in Supplementary Table S1. Quantitative RT–PCR analysis revealed that TERT expression was significantly higher in Kras^mut^ lung adenocarcinomas than in Kras^wt^ lung adenocarcinomas (Figure 6A). As Kras mutations have been associated with cigarette smoking in lung adenocarcinomas, the TERT expression between smokers and nonsmokers was analyzed. We found that smokers had higher TERT levels than non-smokers in lung adenocarcinoma patients (Figure 6B).

## DISCUSSION

In the present study, we found Kras mutations increased TERT expression and telomerase activity and telomere length in both immortalized human bronchial epithelial cells and lung adenocarcinoma cells. However, mutant Kras-induced TERT upregulation is blocked by MEK inhibition. Another study reported that EGF-promoted activation of the RAS-MEK pathway enhances TERT transcription [20]. Kras mutations also cause RAS-MEK pathway activation. We believe that Kras mutations transactivate TERT expression through the activation of RAS-MEK pathway, thereby increasing telomerase activity and telomere length. Since pancreatic tumors and colon tumors which also have a very high percentage of mutant Kras, the telomerase activities of Bx-PC3 pancreatic cancer cells and Caco-2 colon cancer cells with wild-type Kras and Kras^G12D^ and Kras^G12V^ overexpression were tested. We found Kras mutations also increased the telomerase activities in both Bx-PC3 and Caco-2 cells (Supplementary Figure S3), suggesting the generality of Kras mutations transactivating TERT expression in tumor cells.

*In vitro* and *in vivo* experiments have verified that telomerase inhibitors can repress telomerase activities and lead to arrest of tumor growth, reduction of colony formation or prevention of metastases without acute cytotoxicity [23–27]. Telomerase inhibitor BIBR1532, a synthetic, non-nucleoside compound [28, 29], indeed inhibited cell proliferation and transformation in wild-type Kras expressing cells. Importantly, we found that telomerase inhibition suppressed mutant Kras-induced lung carcinogenesis. Although telomerase inhibitors have shown anti-cancer effect on various telomerase-positive cancers, several clinical trials found that overall survival was not improved [30, 31]. Possibly, telomerase inhibitor therapy is only suitable for some cancer subtypes. As a downstream effector of mutant Kras signaling, targeting telomerase could be one potentially promising approach for Kras-mutant NSCLC.

Our results also show telomerase inhibitor significantly increased chemosensitivity of Kras-mutant lung cancer cells. The Kras^G12D^-Calu-3 xenograft mouse model further confirmed that telomerase inhibitor BIBR1532 potentiated the killing effect of paclitaxel by decreasing cell proliferation and increasing cell apoptosis. Considering that many patients with Kras-mutant NSCLC fail to benefit from chemotherapy, combination telomerase inhibitor with chemotherapy may be an effective regimen for the treatment of Kras-mutant NSCLC. We find patients with Kras mutations have higher TERT levels than those with wild-type Kras through examing the surgical specimens from lung adenocarcinoma patients. Compared with nonsmokers, TERT expression is higher in smokers. Previous studies have domenstrated that Kras mutations are associated with smoking status in lung adenocarcinomas [32, 33]. Further investigation is needed to study the correlation of smoking status and Kras mutations and telomerase activity.

Although patients with lung adenocarcinoma that harbors EGFR mutations benefit from EGFR-TKI therapy, Kras mutations appear to confer intrinsic resistance to EGFR-TKIs [34–36]. Interestingly, we found resistance of PC9 cells with Kras^G12D^ mutation and EGFR mutation to EGFR-TKIs was accentuated after telomerase inhibitor treatment (Supplementary Figure S4). Thus, combined telomerase inhibitor and EGFR-TKI may be a useful therapeutic strategy for Kras-mutant NSCLC.

In conclusion, we found Kras mutations increased telomerase activity and lengthened telomeres in NSCLC. Inhibition of telomerase suppressed Kras mutations-driven lung tumorigenesis. Moreover, telomerase inhibitor enhanced the killing effect of paclitaxel *in vivo*. Patients with Kras-mutant NSCLC had higher levels of TERT. Therefore, telomerase inhibitors could be prospectively applied to the treatment of Kras-mutant NSCLC.

## MATERIALS AND METHODS

### Cell lines and cell culture

All cell lines were purchased from the Cell Bank of the Chinese Academy of Sciences (Shanghai, China) where they were tested and authenticated by short tandem repeat and were maintained at 37°C in a 5% CO_2_-humidified atmosphere. Calu-3 cells were cultured in modified Eagle's medium supplemented with 10% fetal bovine serum (FBS) and penicillin/streptomycin (Gibco). BEAS-2B cells were cultured in bronchial epithelial growth medium (CC-3170; Clonetics) supplemented with 10% FBS and penicillin/streptomycin.

Kras, Kras^G12D^ and Kras^G12V^ cDNA were cloned into the pCDH lentiviral expression vector (Systems Biosciences, Mountain View, CA, USA). All the sequences were confirmed by direct nucleotide sequencing. Kras, Kras^G12D^ and Kras^G12V^ were delivered into BEAS-2B and Calu-3 cells by lentivirus infection. The cells were then selected with 2 ug/ml puromycin for 2 weeks, and polyclonal populations were expanded. Telomerase inhibitor BIBR1532 and MEK inhibitor trametinib were purchased from Selleck Chemicals. MEK1 siRNA were purchased from Cell Signaling Technology.

### Quantitative RT–PCR

Expression of TERT mRNA was determined by real-time RT–PCR. Total RNA was extracted using TRIzol (Invitrogen) according to the manufacturer's instructions. cDNA was synthesized using equal amounts of RNA with the PrimeScript II 1st Strand cDNA Synthesis Kit (Takara). Real-time PCR was performed with SYBR Premix Ex Taq II (Takara) in a Bio-Rad CFX96 system (Bio-Rad). Reactions for each sample were performed in duplicate, and the experiment was repeated three times. Results are expressed as relative mRNA levels normalized to GAPDH. Primer sequences are as follows: hTERT:forward, 5′GCCTTCAAGAGCCACGTC3′, reverse, 5′CCACGAAC TGTCGCATGT3′; GAPDH:forward,:5′GGGAAACTGTG GCGTGAT3′,reverse:5′GAGTGGGTGTCGCTGTTGA3

### Western blotting

Cells were lysed in RIPA lysis buffer (1% TritonX-100, 0.1% SDS, 50 mM Tris PH 7.5, 150 mM NaCl, 0.5% sodium deoxycholate, 10 mM NaF) supplemented with protease inhibitor cocktail (Roche) and phosSTOP phosphatase inhibitors (Roche). Cell lysates were fractionated on SDS-PAGE gel and transferred to PVDF membranes (Millipore). Antibodies against KrasG12D, MEK, phospho-MEK, ERK, phospho- ERK, caspase-3, β-actin were purchased from Cell Signaling Technology, and Kras, GAPDH were purchased from Abcam.

### Telomerase activity assay

Real-time quantitative telomeric repeat amplification protocol assays were used to detect telomerase activity [37]. Cells were lysed in CHAPS lysis buffer (Chemicon). The reaction mixtures were cell extracts (~1000 cells), 0.1 ug of telomerase primer TS, and 0.05 ug of anchored return primer ACX, SYBR Green PCR Master Mix (Applied Biosystems). Reaction mixtures were then incubated for 20 min at 25°C, 10 min at 95°C for telomerase inactivation and Taq polymerase activation and amplified in 35 two-step PCR cycles consisting of 30 s at 95°C and 90 s at 60°C. Relative telomerase activity of samples was quantified by determining threshold cycle values (Ct) from semi-log amplification plots (log increase in fluorescence versus cycle number) and comparison to a standard curve generated from serial dilutions of the positive control sample. Standards and negative control were assayed anew on every plate.

### Telomere length assay

Cells were collected at the indicated population points and used for telomere length measurements by the telomere repeat fragment Southern technique as previously described [38].

### Colony formation assay

We seeded 500 cells into each well of 6-well plates in complete medium, using triplicate wells. After 2 weeks of growth, cells were fixed and stained with Giemsa; visible colonies of more than 50 cells were counted.

### Soft agar assay

Viable cells were suspended in 0.3% agarose in medium at a density of ~50,000 cells/well, and plated on a 0.5% agarose base. Cultures were maintained at 37°C in the incubator for 3 weeks. The colonies were stained with 0.5% crystal violet and the number of anchorage-independent colonies was counted in five random fields.

### Cell viability

Approximately 8000 cells were seeded in a 96-well plate, and then exposed to BIBR1532, cisplatin or paclitaxel. A MTS assay was conducted according to the manual (Promega). Experiments were done in triplicate.

### Cell motility assays

Equal numbers of cells were plated in 6-well plates; wounds were generated when the cells reached 90% to 95% confluency with a sterile pipette. Wounds were photographed at various time points with a phase-contrast microscope (Olymbas). A Transwell migration was assessed using 24-well Transwell polycarbonate filters (Corning Costar Corp.) with an 8-um pore size. Approximately 50,000 cells were seeded onto each upper chamber of 24-well Transwell plates and incubated in serum-free medium. Medium containing 10% FBS was placed in the bottom chamber and served as a chemoattractant. Eight or twelve hours later, cells on the upper surface of the filter were removed by gently wiping with a cotton swab. Cells that migrated to the bottom of the filter were fixed and stained with crystal violet. Migrated cells were visualized by microscope and counted in at least five random fields.

### Tumor growth assay

Calu-3-Kras^G12D^ cells (4 × 10^6^ cells) were injected s.c. into the flanks of 5-week-old female BALB/c nude mice (*n* = 24). Mice were randomly divided into four groups at 9 days after implantation and intraperitoneally injected with BIBR1532 and paclitaxel for short-term treatments. Tumor growth was measured with a caliper every 3 days and calculated as (length × width^2^)/2. At 5–7 weeks after transplantation, the tumors were isolated and fixed in 10% buffered formalin and embedded in paraffin. Tumor sections were stained for hematoxylin and eosin. Ki-67 (1:200 dilution, Dako) and cleaved caspase-3 (1:50 dilution, Cell Signaling Technology) expression were detected by Immunohistochemistry. The animal protocol was reviewed and approved by the Institutional Animal Care and Use Committee of Tianjin Cancer Hospital.

### Patient and tissue samples

We obtained frozen tissue samples from 22 patients with primary lung adenocarcinoma who underwent definitive surgical treatment between 2014 and 2015 at Tianjin Cancer Hospital. Kras mutations were detected by the amplification refractory mutation system (ARMS) assay. None of the patients received chemotherapy or radiotherapy before operation. Their clinico-pathological characteristics are summarized in Supplementary Table S1. This study was approved by the Institutional Review Board of Tianjin Cancer Hospital.

### Statistical analyses

Statistical analysis was performed with GraphPad Prism 6 software (GraphPad software, CA, USA). Data from *in vitro* and *in vivo* experiments are presented as the mean ± standard error (SEM) and were assessed by one-way ANOVA. Differences were considered significant when the *p* value was less than 0.05.

## SUPPLEMENTARY MATERIALS


