# Supplementary material for: Kras mutations increase telomerase activity and targeting telomerase is a promising therapeutic strategy for Kras-mutant NSCLC
Source: Oncotarget. 2016 Jun 18;8(1):179–90. doi: 10.18632/oncotarget.10162 (PMC5352098; doi:10.18632/oncotarget.10162)
Supplement: Supplementary file 1 [file oncotarget-08-179-s001.pdf]

Kras mutations increase telomerase activity and targeting telomerase is a promising therapeutic strategy for Kras-mutant NSCLC

Supplementary Materials

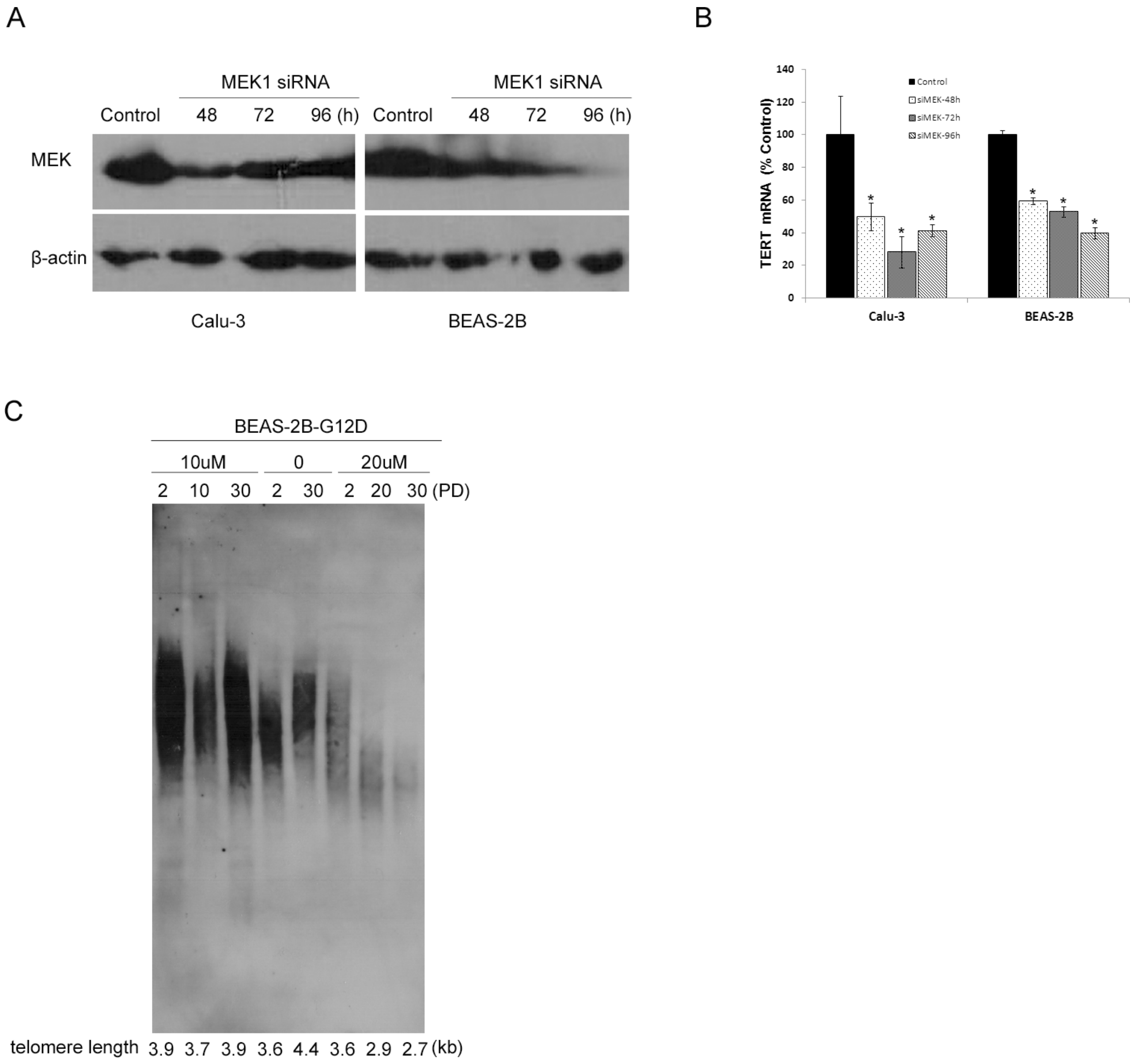

Supplementary Figure S1: MEK siRNA-mediated MEK knockdown reduces TERT mRNA levels in Kras^{G12D}-overexpressing cells. (**A**) Kras^{G12D} -overexpressing cells were transfected by MEK siRNA. The inhibitory activity of MEK siRNA was examined by immunoblot analysis with antibody against to MEK. (**B**) TERT mRNA expression was analyzed by RT-qPCR (*$P < 0.05$). (**C**) KrasG12D -overexpressing BEAS-2B cells were treated with different concentrations of BIBR1532. TRF Southern blot analysis showed that 10 micromoles of BIBR1532 treatment did not shorten telomeres, whereas 20 micromoles significantly shortened telomeres.

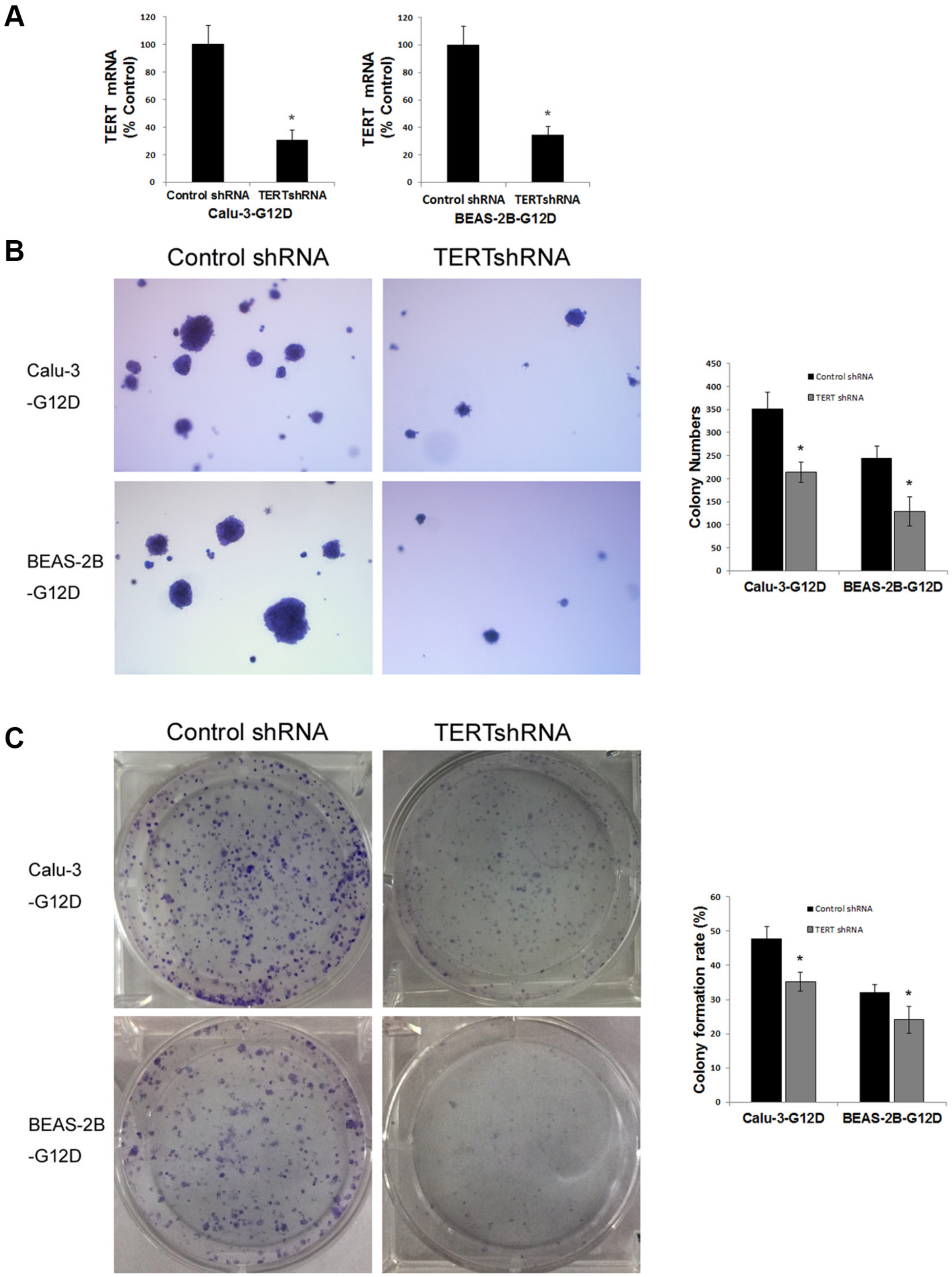

Supplementary Figure S2: shRNA-mediated knockdown of TERT inhibits anchor-independent growth and clone formation in Kras^{G12D}-overexpressing cells. (**A**) Lentiviral vectors expressing TERT shRNA were delivered into Kras^{G12D} -overexpressing cells to knock down the expression of TERT. (**B**) TERT downregulation inhibited anchor-independent growth in soft agar. (**C**) TERT downregulation inhibited clone formation (*P < 0.05).

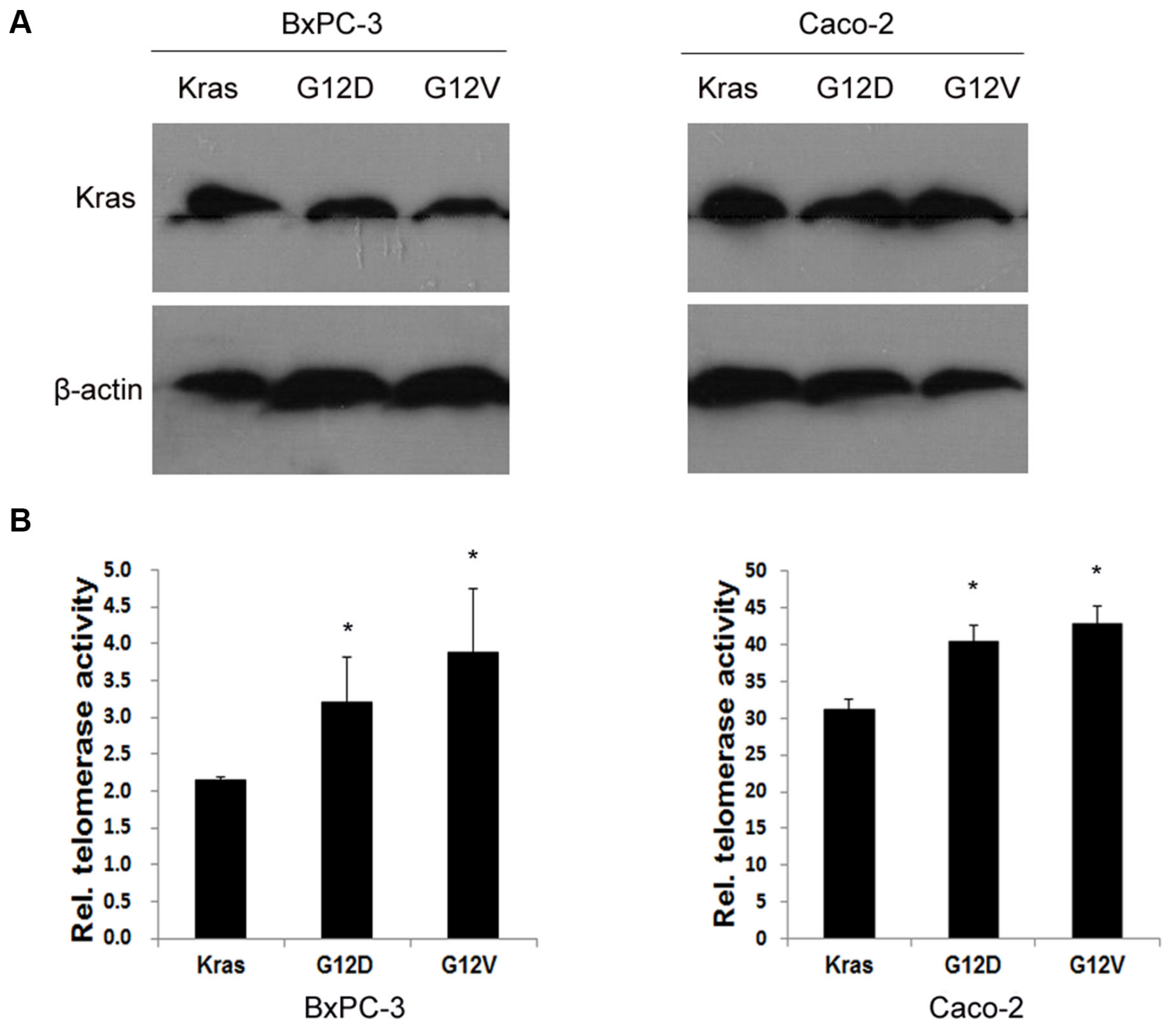

Supplementary Figure S3: Kras mutations increased telomerase activities in Bx-PC3 pancreatic cancer cells and Caco-2 colon cancer cells. (**A**) Wild type Kras, $Kras^{G12D}$ and $Kras^{G12V}$ were lentivirally transduced into Bx-PC3 and Caco-2 cells. Kras expression was tested by immunoblot analysis with antibody against to pan- Kras. (**B**) Telomerase activity was examined by a real-time quantitative TRAP assay (*$P < 0.05$).

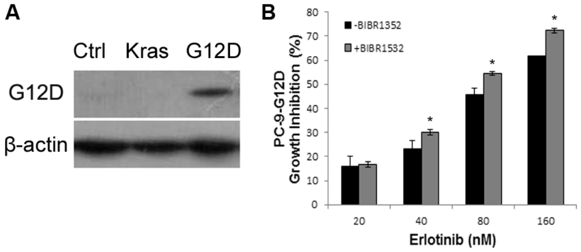

Supplementary Figure S4: Telomerase inhibitor BIBR1532 enhances the sensitivity of $Kras^{G12D}$ –overexpressed PC-9 cells to erlotinib. (**A**) PC-9 cells were transduced by lentiviruses expressing $Kras^{G12D}$ cDNA. Immunoblot analysis of $Kras^{G12D}$ expression with $Kras^{G12D}$ mutant-specific antibody. (**B**) $Kras^{G12D}$-overexpressed PC-9 cells were treated with erlotinib or erlotinib plus BIBR1532 (20 uM), cell viability was measured 72 hours after treatment. Data were expressed as mean ± SEM from three independent experiments (*$P < 0.05$).

Supplementary Table S1: Clinicopathological Details of 22 Lung Adenocarcinomas Patients

Patient No.	Age	Gender	Tumor size (cm)	TNM stage	Smoking status	Kras
1	55	F	5	I B	Nonsmoker	Mutant-type
2	59	F	3	I A	Smoker	Mutant-type
3	60	M	2	III A	Smoker	Mutant-type
4	68	F	6	III A	Nonsmoker	Mutant-type
5	45	M	2.5	III A	Smoker	Mutant-type
6	72	M	12	III A	Smoker	Mutant-type
7	65	F	5	II A	Smoker	Mutant-type
8	63	M	4	II A	Smoker	Mutant-type
9	73	M	9	IV	Smoker	Mutant-type
10	51	M	6	III A	Smoker	Mutant-type
11	63	M	5	III A	Smoker	Mutant-type
12	64	M	8	II B	Smoker	Wild-type
13	56	F	3	I A	Nonsmoker	Wild-type
14	64	F	6	II A	Nonsmoker	Wild-type
15	59	M	3.5	III B	Smoker	Wild-type
16	59	F	5.5	III A	Nonsmoker	Wild-type
17	69	M	6.5	II A	Smoker	Wild-type
18	75	M	3	III A	Nonsmoker	Wild-type
19	62	F	2.5	III A	Smoker	Wild-type
20	31	F	3	II A	Nonsmoker	Wild-type
21	47	M	3.5	III A	Smoker	Wild-type
22	47	M	5	III A	Nonsmoker	Wild-type
